# Assessment of genome integrity with array CGH in cattle transgenic cell lines produced by homologous recombination and somatic cell cloning

**DOI:** 10.1186/2041-9414-2-6

**Published:** 2011-05-23

**Authors:** George E Liu, Yali Hou, James M Robl, Yoshimi Kuroiwa, Zhongde Wang

**Affiliations:** 1 USDA-ARS, ANRI, Bovine Functional Genomics Laboratory, Beltsville, Maryland 20705, USA; 2 Department of Animal and Avian Sciences, University of Maryland, College Park, Maryland 20742, USA; 3 Hematech Inc, Sioux Falls, SD, 57106, USA; 4 48351, 277th Street, Canton, SD, 57013, USA

**Keywords:** genome integrity, cattle transgenic cell line, somatic cell cloning, array CGH

## Abstract

**Background:**

Transgenic cattle carrying multiple genomic modifications have been produced by serial rounds of somatic cell chromatin transfer (cloning) of sequentially genetically targeted somatic cells. However, cloning efficiency tends to decline with the increase of rounds of cloning. It is possible that multiple rounds of cloning compromise the genome integrity or/and introduce epigenetic errors in the resulting cell lines, rendering a decline in cloning. To test these possibilities, we performed 9 high density array Comparative Genomic Hybridization (CGH) experiments to test the genome integrity in 3 independent bovine transgenic cell lineages generated from genetic modification and cloning. Our plan included the control hybridizations (self to self) of the 3 founder cell lines and 6 comparative hybridizations between these founders and their derived cell lines with either high or low cloning efficiencies.

**Results:**

We detected similar amounts of differences between the control hybridizations (8, 13 and 39 differences) and the comparative analyses of both "high" and "low" cell lines (ranging from 7 to 57 with a mean of ~20). Almost 75% of the large differences (>10 kb) and about 45% of all differences shared the same type (loss or gain) and were located in nearby genomic regions across hybridizations. Therefore, it is likely that they were not true differences but caused by systematic factors associated with local genomic features (e.g. GC contents).

**Conclusions:**

Our findings reveal that large copy number variations are less likely to arise during genetic targeting and serial rounds of cloning, fortifying the notion that epigenetic errors introduced from serial cloning may be responsible for the cloning efficiency decline.

## Findings

As embryonic stem cells are not available in the bovine species, somatic cells have been used for genetic modifications, and transgenic cattle have been produced from such genetically modified somatic cells by animal cloning. However, because primary somatic cells have limited life span and inevitably become senescent following DNA transfection and selection in cell culture, it is impossible to perform any further genetic modifications in these cells. Because of such, transgenic cattle with a desired genotype that requires more than one genetic targeting event, such as homozygous deletion of the two alleles of a gene, cannot be produced. To overcome such limitations, a novel sequential genetic modification strategy in bovine somatic cells, for producing extensively genetically modified cattle, has been developed [1]. This process involves a serial round of genetic targeting events, each followed by cloning to rejuvenate the genetically modified somatic cells (to rescue them from senescence) for the next round of genetic targeting. Such genetically modified somatic cells are then subjected to a final round of cloning for producing transgenic animals with the desired genotypes. While multiple genomic loci have been modified by this strategy, cloning efficiency tends to decline with the increased rounds of cloning, and in some severe cases, such manipulated cells can become unclonable (no live calf can be cloned from them) [2]. It is yet unknown whether the cloning efficiency declines in such derived cells are due to genetic abnormalities caused by the multiple genetic targeting or/and serial cloning process or due to the accumulation of epigenetic errors introduced during these processes. Such questions are fundamental in farm animal transgenesis, as somatic cells and cloning are currently the only choices for genetic modifications and for transgenic animal production in the domestic animal species.

To investigate whether the declines of cloning efficiency in the cloned bovine transgenic cell lines are due to large genomic deletions or insertions, 9 high density array Comparative Genomic Hybridization (CGH) experiments were performed to test the genome integrity in 3 independent bovine transgenic cell lineages. Array CGH allows the entire genome to be assayed for the gain or loss of material in a single experiment by measuring the relative hybridization intensity between fluorescently labeled test and reference DNA samples. It has been widely used in the detection of copy number variations (CNVs). One objective of this study is to develop array CGH into a systematic test for the genomic integrity of donor cells after each round of genetic modification before they are used as donors for producing transgenic animals.

**Figure 1:**
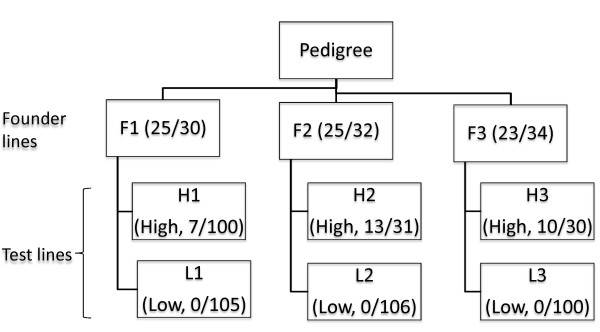
**Three cell lineages (founders and test cell lines) and their success rates for animal cloning**. Live calving rates for the cell lines were calculated by the live calf counts at birth divided by recipient numbers used for embryo transfer as shown in parentheses. Cell lines with 7% or more living rates are indicated as High (H; high calving rate) and those with 0% live calving rate as Low (L; low calving rate). The 3 founder cell lines (F1, F2 and F3) were established from 3 different fetuses (day 40) respectively that were produced by artificial insemination. The 6 test cell lines, except for cell line L3, were derived from 2 rounds genetic modification and somatic cell cloning. L3 line was derived from 3 rounds of genetic modification and somatic cell cloning.

We selected 3 independent cell lineages from our transgenic bovine cell line collection. Each lineage includes the founder and two derived cell lines, which demonstrated dramatic differences in cloning efficiency (Figure 1). The cloning efficiencies are represented by the live calf counts at birth divided by recipient numbers used for embryo transfer as shown in parentheses. Test lines were classified into "high (H)" and "low (L)" based on their cloning efficiencies, with 7%-42% live calving rates designated as high and 0% as low. The procedures for genetic modifications, animal cloning and transgenic cell line establishment were described previously [1]. Genomic DNA samples were purified from the cell lines using Qiagen Miniprep Kit as recommended by the manufacturer. All DNA samples were analyzed by Nanodrop spectrophotometer and agarose gel electrophoresis. Nine array CGH experiments were carried out using each cell line as the test sample and the corresponding founder line as the reference sample (Table 1). Therefore, our plan included the control hybridizations of the 3 founder cell lines (self to self) and 6 comparative hybridizations between these founders and their derived cell lines of extreme phenotypes ("high" versus "low" cloning efficiencies). Another self to self control hybridization was performed using the sequenced Hereford cow L1 Dominette 01449 (Dt, American Hereford Association registration number 42190680). Each CGH array contains ~2.1 million oligonucleotide probes that provide a genome-wide coverage with an average interval of ~1.2 kb (kilo basepairs) on the UMD3 genome assemblies [3]. DNA labeling, hybridizations, array scanning, data normalization, and segmentation were performed as described before [4,5]. The genomic variations were represented by gains and losses of normalized fluorescence intensities relative to the reference. The calls are filtered according to the similar criteria as described previously [6]. Briefly, we tested a series of log2 ratio shift and affected neighboring probe counts and their impact on the false discovery rate in the self-self control hybridizations. We then selected a calling criterion, requiring that alternations of 0.5 log2 ratios over 5 neighboring probes, under which minimal false positives were found for self-self control hybridizations. Thus, the arrays have a resolution of approximately 4.8 kb. Nine array CGH data have been submitted to the gene expression omnibus (http://www.ncbi.nlm.nih.gov/geo/) under the accession number GSE26132.

**Table 1:** Hybridization plan and event counts

No	Test	Ref	Type	Events
1	F1	F1	Self1	13
2	H1	F1	High1	13
3	L1	F1	Low1	7
4	F2	F2	Self2	8
5	H2	F2	High2	7
6	L2	F2	Low2	57
7	F3	F3	Self3	39
8	H3	F3	High3	17
9	L3	F3	Low3	22
10	Dt	Dt	Self4	3

**Figure 2:**
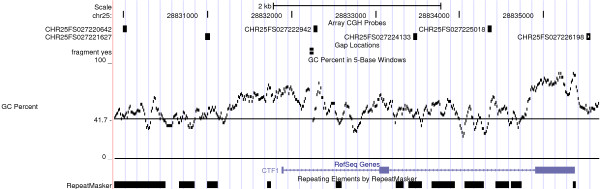
**False positive event calls could be due to high GC content**. A 5.5 kb variable region (chr25:28829889-28835660) was identified in one control self to self array CGH. GC Percent in 5-Base Windows, Array CGH probe, Gap, RefSeq Gene and Repeat tracks are displayed in Btau_4.0. The GC percent track shows the percentage of G (guanine) and C (cytosine) bases in 5-base windows. The horizontal line at 41.7 in GC percent track represents the genome average of GC%. Probe locations are labeled like CHR25FS027220642 and etc.

We detected 8, 13 and 39 differences in 3 control hybridizations. Similar amounts of differences (ranging from 7 to 57 with a mean of ~20) were detected in comparative analyses of both "high" and "low" derived cell lines (Table 1 and Table 2). We also made event calls on Btau_4.0 and obtained a comparable number of events (data not shown). Almost 75% of the large differences (>10 kb, 42/58 events in Table 2) and about 45% of all differences (82/186 events) shared the same type (loss or gain) and were located in nearby genomic regions across hybridizations. Therefore, it is likely that they were not true differences but instead caused by systematic factors like dye bias (Cy3 versus Cy5) or genomic waves associated with local genomic features, such as GC contents [7]. For example, a variable region of chr25:27220643-27226199 from UMD3 (5.5 kb) was found in hybridizations of High1, Self3 and High3. Using liftOver, we migrated this region to its corresponding region at chr25:28829889-28835660 on Btau_4.0. The GC% track and array CGH probe track are shown in the UCSC genome browser snapshot (Figure 2). Although each probe has a GC% range from 42-48%, the average GC% of this region (53.5%) is significantly higher than the cattle genome average of 41.7% and multiple GC% peaks exist in the close proximity of 3 out of the 6 probes. Out of 186 events, 129 events are unique after merging the overlapped events (data not shown). Out of these 129 unique events, 71 events can be successfully migrated from UMD3 to Btau_4.0 and all of them showed various degrees of higher GC contents as compared to the genome average.

**Table 2:** Copy number variation events larger than 10 kb

No	Type	Chr	Start	End	Length	Log R	Shared
1	Self1	chr13	48,998,999	49,016,999	18,000	0.5168	Yes
		chr3	1,020,294	1,039,699	19,405	0.6721	Yes
		chr4	41,465,452	41,496,569	31,117	0.6946	Yes
2	High1	chr13	48,992,999	49,010,999	18,000	0.6269	Yes
		chr3	1,020,294	1,039,699	19,405	0.6410	Yes
		chr4	33,570,495	33,584,300	13,805	0.5216	Yes
		chr4	41,465,452	41,496,569	31,117	0.5674	Yes
3	Low1	chr13	48,991,360	49,017,997	26,637	0.6818	Yes
		chr29	19,400,430	19,449,274	48,844	0.5343	Yes
		chr3	1,020,294	1,042,839	22,545	0.7566	Yes
		chr4	41,465,452	41,496,569	31,117	0.8130	Yes
4	Self2	chr13	48,991,360	49,017,997	26,637	0.5172	Yes
		chr3	1,020,294	1,042,839	22,545	0.6039	Yes
		chr4	41,465,452	41,496,569	31,117	0.5761	Yes
5	High2	chr25	32,373,045	32,464,814	91,769	0.6769	Yes
6	Low2	chr11	87,532,580	87,543,090	10,510	-0.5617	No
		chr2	16,958,057	16,968,620	10,563	-0.5814	No
		chr25	32,374,157	32,471,634	97,477	-0.8136	Yes
		chr29	43,204,051	43,223,301	19,250	-0.5334	No
		chrX	10,447,331	10,457,486	10,155	-0.9137	No
7	Self3	chr1	5,249,999	5,285,999	36,000	0.5481	Yes
		chr10	59,478,526	59,531,204	52,678	0.5501	No
		chr13	48,991,360	49,017,997	26,637	0.6809	Yes
		chr15	26,576,999	26,602,199	25,200	0.6474	Yes
		chr2	39,223,655	39,235,168	11,513	-0.5661	No
		chr25	32,374,157	32,471,634	97,477	-0.6113	Yes
		chr29	19,399,250	19,449,274	50,024	0.6094	Yes
		chr3	1,020,294	1,039,699	19,405	0.8678	Yes
		chr4	27,707,990	27,750,008	42,018	0.5614	No
		chr4	41,465,452	41,496,569	31,117	0.8426	Yes
		chr6	45,738,703	45,776,348	37,645	0.5049	Yes
		chr6	89,209,799	89,220,599	10,800	0.6477	Yes
		chr8	36,073,799	36,145,799	72,000	0.5122	Yes
		chrX	37,290,568	37,303,155	12,587	0.8150	No
		chrX	37,564,199	37,614,599	50,400	0.5427	No
		chrX	56,120,456	56,149,298	28,842	0.6124	No
		chrX	84,230,177	84,255,543	25,366	0.5679	No
		chrX	138,374,999	138,386,999	12,000	0.5528	No
8	High3	chr13	48,993,325	49,013,328	20,003	0.5149	Yes
		chr15	26,576,999	26,602,199	25,200	0.6022	Yes
		chr25	32,374,157	32,403,637	29,480	-0.8278	Yes
		chr4	33,564,599	33,578,999	14,400	0.5363	Yes
		chr4	41,466,599	41,495,399	28,800	0.5097	Yes
		chr6	45,744,065	45,772,999	28,934	0.5284	Yes
9	Low3	chr1	5,249,999	5,285,999	36,000	0.5559	Yes
		chr1	144,107,850	144,130,905	23,055	0.5949	No
		chr13	48,991,360	49,017,997	26,637	0.6031	Yes
		chr17	73,139,605	73,159,081	19,476	-2.0603	No
		chr18	6,080,815	6,121,152	40,337	-0.5749	No
		chr25	32,362,844	32,470,747	107,903	0.6669	Yes
		chr29	19,412,812	19,444,215	31,403	0.6690	Yes
		chr3	1,020,599	1,038,599	18,000	0.6787	Yes
		chr4	33,564,599	33,578,999	14,400	0.6100	Yes
		chr4	41,465,452	41,487,890	22,438	0.6229	Yes
		chr6	89,208,198	89,218,288	10,090	0.6937	Yes
		chr8	36,077,399	36,152,999	75,600	0.5089	Yes
		chrU	12,620,478	12,665,758	45,280	0.7441	No
10	Self4	chr13	48,992,999	49,010,999	18,000	0.5729	Yes

Chr: chromosome, Log R: log2Ratio, Shared: Yes/No - events shared among samples (i.e. hybridizations) or not.

In this project, we employed array CGH to study genomic integrity in cattle transgenic cell lines. This high-resolution genome-wide survey fills the knowledge gaps left out in the existing literature. Our results generate a valuable tool for genomic integrity evaluation and largely exclude the occurrences of large genomic structural variations (≥ 10 kb) during animal cloning, supporting our recent findings that epigenetic errors introduced by multiple rounds of cloning and/or genetic targeting are the possible underlying causes for the cloning efficiency decline [8,9]. However, this initial genomic integrity survey reported here is probably not complete as the CGH arrays were designed by using only one reference genome. As a result, sequences absent in Dominette and present in other animals cannot be ascertained. Also, array CGH cannot detect small event (<5 kb) and balanced events like inversions and translocations. Therefore, we cannot totally exclude the possibility that both genetic and epigenetic influences may be at work and genetic differences may have played a role in the low efficiencies. With the costs of genome sequencing dropping dramatically by using next-generation sequencing, emerging high-quality cattle genomic sequence will soon facilitate the application of the direct sequence comparison strategy. Furthermore, additional studies like epigenomics are warranted and may unravel the epigenetic basis for the successful and efficient animal cloning.

## Competing interests

YK and ZW are employees of Hematech, Inc., a subsidiary of Kyowa Hakko Kirin Company, Ltd. The authors declare that they have no competing interests.

## Authors' contributions

GEL and ZW conceived and designed the experiments. JMR provided reagents. GEL and YH performed *in silico *prediction and computational analyses. GEL, YK and ZW wrote the paper.

All authors have read and approved the final manuscript.
